# Diabetes Is a Risk Factor for the Prognosis of Patients with Bladder Cancer: A Meta-Analysis

**DOI:** 10.1155/2022/1997507

**Published:** 2022-09-26

**Authors:** Lv Dong, Xiang Ying, Song Tao, Zhou Guang-Peng, Shen Tai-Ming

**Affiliations:** ^1^Department of Urology, Deyang People's Hospital, Deyang, Sichuan, China; ^2^Department of Urology, Eastern Hospital, Sichuan Academy of Medical Sciences &Sichuan Provincial People's Hospital, Chengdu, Sichuan, China; ^3^Department of Endocrinology, Eastern Hospital, Sichuan Academy of Medical Sciences &Sichuan Provincial People's Hospital, Chengdu, Sichuan, China; ^4^Health Management Center, Sichuan Academy of Medical Sciences and Sichuan Provincial People's Hospital, Chengdu, Sichuan, China

## Abstract

**Objective:**

To systematically evaluate the impact of diabetes on the prognosis of bladder cancer patients after radical cystectomy (RC).

**Methods:**

PubMed, Embase, and Cochrane Library databases were selected from inception to October 2021. The studies on the effects of diabetes on bladder cancer patients after RC were included for analysis. The inclusion and exclusion criteria were independently selected for literature screening, the quality of the included studies was evaluated, and data were extracted.

**Results:**

A total of 5 cohort studies were included, with a total of 2 661 subjects, including 391 cases in the diabetic group, non-diabetes. Meta-analysis results show that diabetes increases the overall risk of death in patients after RC (HR = 1.36, 95% CI: 1.30 ∼ 1.43, *P* < 0.001) and the risk of tumor-specific death (HR = 1.59, 95% CI: 1.29 ∼ 1.95, *P* < 0.001). Sensitivity analysis shows that the stability of this study is well.

**Conclusion:**

Diabetes was an independent risk factor in terms of overall and cancer-specific survival in patients who underwent RC.

## 1. Introduction

Bladder cancer is one of the most common malignant tumors of the urinary tract, and its incidence is higher in men than in women [1]. More than 80% of bladder cancer patients will experience painless hematuria or symptoms of overactive bladder such as frequent urination and urgency [2, 3]. Although bladder cancer is not among the top ten causes of cancer death in China, it is one of the most common malignant tumors in urinary tract diseases. The incidence of bladder cancer ranks 10th among malignant tumors in the world. About 80,000 new bladder cancer patients were diagnosed in the United States in 2017, while the age-standardized incidence rates of bladder cancer in European men and women reached 19.1 and 4.0, respectively. About 30% of all newly diagnosed bladder cancer patients have muscle-invasive bladder cancer (MIBC), and the standard treatment is radical cystectomy [4, 5]. Although MIBC has made some progress in the treatment in recent years, the overall prognosis remains poor.

Diabetes is the most common chronic metabolic disease in the world, and it may have an important impact on the pathogenesis, progression, and prognosis of bladder cancer through various mechanisms [6, 7]. The paper will discuss the effect of diabetes on the survival of patients with bladder cancer after RC surgery and provide evidence for improving the prognosis of patients with bladder cancer.

## 2. Methods

### 2.1. Literature Review

The databases including PubMed, Embase, and Cochrane Library were all screened for related studies from their inception to November 2021. The search terms included “diabetes,” “bladder cancer,” “urothelial carcinoma,” “bladder neoplasms,” “urinary bladder neoplasms,” “cystectomy,” “mortality,” “survival,” and “prognosis.”

### 2.2. Inclusion Criteria

Inclusion criteria were as follows: (1) a cohort study with bladder cancer patients as the research object; (2) pathologically diagnosed patients with high-risk non-muscle-invasive bladder cancer or muscle-invasive bladder cancer, and the selected treatment method is RC; (3) in the diabetes group, the diagnosis of diabetes should be before RC; (4) outcome indicators include overall survival (OS) or cancer-specific mortality and other related prognostic indicators; (5) the hazard ratio (HR) and confidence interval (95%CI) are provided in the literature or the HR and 95% CI can be extracted from the Kaplan–Meier curve in the literature; and (6) if the same study has published results from different periods, the latest research data will be included.

### 2.3. Exclusion Criteria

Exclusion criteria were as follows: (1) epidemiological investigation, case-control study, and other research types; (2) research using partial cystectomy or transurethral resection of bladder tumor and other methods of treatment; (3) literature types such as review, medical record report, meta-analysis, conference paper, and so on; (4) studies with poor research quality and without full text; and (5) studies without reporting relevant outcome indicators.

### 2.4. Literature Quality Evaluation

Two reviewers independently screened the literature and evaluated the quality of the literature according to the inclusion and exclusion criteria and then cross-compared the extracted study data. The Newcastle-Ottawa Scale (NOS) cohort study scoring table was used to evaluate the quality of the literature, and the literature with a score of ≥6 was considered as high-quality literature and could be included in meta studies.

### 2.5. Data Extraction

The data mainly extracted according to the purpose of the study included the first author's name, publication time, country, cohort age, sample size, age, gender, follow-up time, HR, and 95% CI for survival risk or death risk.

### 2.6. Statistical Analysis

Meta-analysis was performed by RevMan software, and the HR and 95% CI in each study were pooled. The *I*^*2*^ test was used to test the heterogeneity of the included studies. If *I*^2^ ≥ 50%, *P* ≤ 0.10, there was heterogeneity among the studies, and a random-effects model was used for analysis; if *I*^2^ < 50%, *P* > 0.10, there was no significant heterogeneity, and a fixed-effects model was used for analysis. Sensitivity analysis was performed on the results of the meta-analysis, and funnel plots were drawn for publication bias analysis. If there was publication bias, the cut-and-fill method was used to verify whether the publication bias affected the stability of the combined effect size.

## 3. Results

### 3.1. Baseline Characteristics of the Included Studies

**Figure 1:**
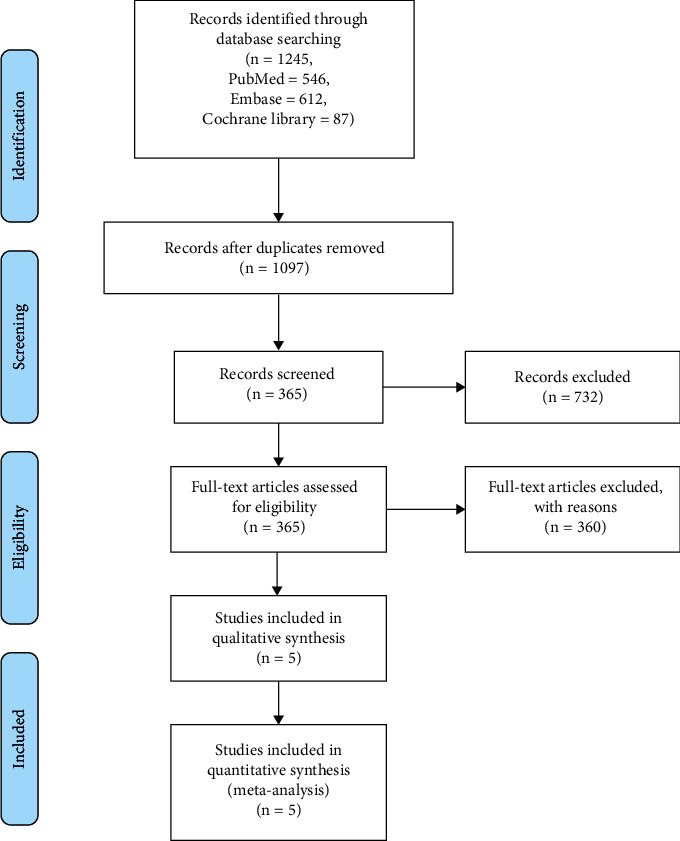
The flowchart of the literature screening.

After the screening of the studies, we got 1245 studies from the databases. After the duplications were excluded, 1097 studies were left. After the assessment of title and the abstract of the studies, 365 studies were left. And we performed the full-text screening, we finally 5 studies in the present analysis [8–12]. The screening procedure is shown in Figure 1. The baseline characteristics of the included studies are presented in Table 1.

### 3.2. Overall Survival

**Figure 2:**
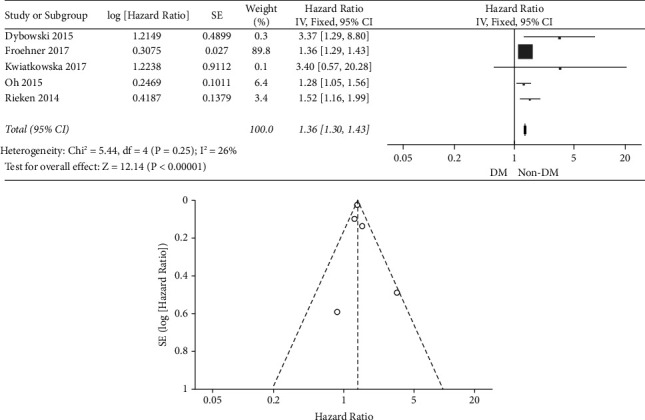
The forest and funnel plots of the overall survival.

As shown in Figure 2, the meta-analysis showed that diabetes was significantly associated with the poor overall survival of the patients (HR = 1.36, 95% CI: 1.30 ∼ 1.43, *P* < 0.001). Also, the funnel plot demonstrated that there was no significant publication bias.

**Figure 3:**
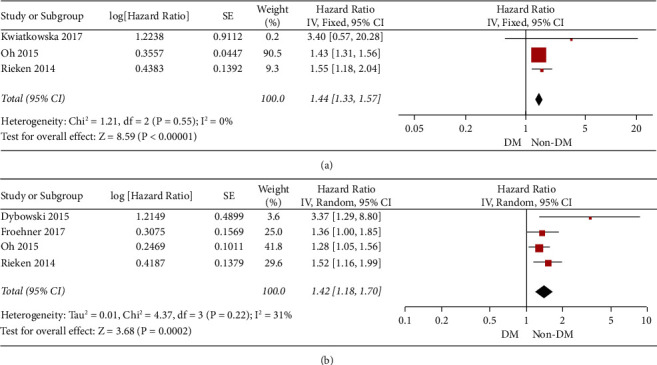
The forest plot for the overall survival in (a) univariate analysis and (b) multivariate analysis.

Then, we performed the subgroup analysis based on the analysis type of the included studies. We first pooled the results from the univariate analysis results. As shown in Figure 3(a), the results in univariate analysis were consistent with the above results. In Figure 3(b), we observed that the diabetes was still significantly associated with the overall survival of the patients in the multivariate analysis.

### 3.3. Cancer-Specific Survival and Recurrence

**Figure 4:**
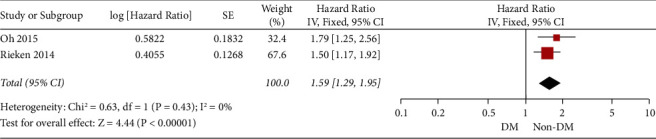
The forest plot for the cancer-specific survival.

**Figure 5:**
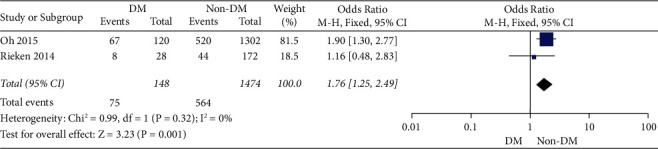
The forest plot for the recurrence rate.

We studied cancer-specific survival next. As shown in Figure 4, diabetes was significantly associated with the cancer-specific survival of the included patients.  Figure 5 shows the results on the recurrence rate. Diabetes was significantly associated with the recurrence of the disease.

## 4. Discussion

Bladder cancer is the most common malignant tumor of the urinary system [13]. In 2018, about 200,000 people died of bladder cancer worldwide, accounting for 2.1% of cancer-related deaths. The most common histological type of bladder cancer is urothelial carcinoma, accounting for about 90% of all bladder cancers [14, 15]. For urothelial cell muscle-invasive bladder cancer and non-urothelial cell bladder cancer, active RC is recommended. The standard treatment for RC is “total cystectomy + distal ureterectomy + pelvic lymph node dissection + urinary diversion.” RC can be open, laparoscopic, or robotic, and each treatment technique has its own advantages and disadvantages. However, RC is a complex surgical procedure, the incidence of postoperative complications can be as high as 64%, the 30-day postoperative mortality rate is 1% to 3%, and the postoperative mortality rate will increase for patients aged >80 years [16].

Diabetes is a chronic metabolic disease. Due to the absolute or relative lack of insulin, the blood sugar in the body remains at a high level for a long time. If it is not effectively controlled, it will eventually lead to cardiovascular and cerebrovascular diseases, kidney damage, retinopathy, and peripheral neuropathy [17]. Diabetes is one of the most serious health problems in the world today. There are 22 million people living with diabetes in the United States, and this number is expected to climb to 40 million by 2030. There are about 69.1 million diabetics in India and 69.1 million in China. The total number of people with diabetes and prediabetes has reached 140 million. There is a close relationship between diabetes and bladder cancer, and many studies have shown that diabetes can increase the incidence and mortality of bladder cancer [18–21].

In this study, meta-analysis confirmed that diabetes can significantly increase the risk of death in bladder cancer patients after RC, resulting in poor prognosis of patients. The remainder of this paper is arranged as the following two reasons why diabetes increases the risk of death in patients after RC surgery. (1) The incidence of complications in diabetic patients is higher. Studies have demonstrated that diabetes is an independent risk factor for perioperative life-threatening complications in cystectomy patients and is positively associated with serious complications. The study by Goodenough et al. pointed out that glycated hemoglobin ≥6.5% and perioperative hyperglycemia were associated with an increased incidence of major complications after abdominal surgery, emphasizing that elective surgery should be more reasonably controlled for blood sugar. Mossanen et al. included 57 553 patients with RC surgery in 360 hospitals in the United States. The meta-analysis showed that the increase in complications will lead to an increase in the mortality rate of patients. (2) Diabetes can lead to tumor recurrence and progression. Type 2 diabetes is the most common type of diabetes, and insulin resistance and hyperinsulinemia often exist in the body. Insulin can combine with the insulin receptor on the surface of tumor cells to promote the mitosis of tumor cells.

There are few studies on the effect of diabetes on the prognosis of patients with RC after surgery, and there is no relevant meta-analysis conducted by scholars at present, so the topic selection of this study is relatively new, and the quality of the included literature is high. However, this study also has some limitations. First, only 5 cohort studies were included, and only 2 studies reported tumor-specific mortality risk. None of the 5 studies analyzed glycemic control after RC. Second, the sample sizes of the two studies were small, so further large-sample prospective studies are still needed for demonstration and analysis. Finally, this study only retrieved published Chinese and English studies and did not include dissertations, conference papers, and unpublished studies. There may be publication bias caused by incomplete literature inclusion. It is proved that the stability and robustness of the results of this meta-analysis are high.

The results of this study prove that diabetes can significantly increase the risk of death after radical cystectomy in bladder cancer patients. For the future improved treatments of bladder cancer patients with diabetes, close follow-up should be performed during the perioperative period and postoperative period to monitor and control blood sugar levels to reduce the risk of related complications.

## 5. Conclusion

Diabetes was an independent risk factor in terms of overall and cancer-specific survival in patients who underwent radical cystectomy.

## Figures and Tables

**Table 1:** The baseline characteristics of the included studies.

Author	Year	Region	Study type	Study period	Sample size		Age	Gender (male/female)	Follow-up	NOS
					DM	Non-DM				
Rieken et al. [12]	2014	USA, Canada, Australia, and Europe	*R*	1992–2008	120	1302	65.5	1108/314	34	8
Oh et al. [11]	2015	Korea	*R*	2004–2014	28	172	65.8	176/24	38	7
Dybowski et al. [8]	2015	Poland	*R*	2004–2006	10	53	67	46/17	81	8
Froehner et al. [9]	2017	Germany	*R*	1993–2012	225	707	68	726/206	84	7
Kwiatkowska et al. [10]	2017	Poland	*P*	2014–2016	8	36	67	25/19	16	8

*Note*. DM: diabetes, *R*: retrospective study, and *P*: perspective study.

## Data Availability

The data used to support this study are available from the corresponding author upon request.
